# Reducing the foot trajectory variabilities during walking through vibratory stimulation of the plantar surface of the foot

**DOI:** 10.1038/s41598-021-86583-7

**Published:** 2021-03-29

**Authors:** Shun Yamashita, Kotaro Igarashi, Naomichi Ogihara

**Affiliations:** 1 Department of Mechanical Engineering, Faculty of Science and Technology, Keio University, Yokohama, 223-8522 Japan; 2 Department of Biological Sciences, Graduate School of Science, The University of Tokyo, 7-3-1 Hongo, Bunkyo-ku, Tokyo, 113-0033 Japan

**Keywords:** Biomedical engineering, Motor control

## Abstract

Variabilities or fluctuations in foot clearance are considered as a risk factor for falls during walking in older adults. The present study aimed to investigate whether the foot trajectory variability can be reduced by applying vibratory stimulation to the foot's plantar surface during walking. Ten healthy adults were asked to walk on a treadmill with vibratory shoes, and body kinematics were measured. Changes in the mean absolute deviations of the foot trajectory and joint and trunk angles were compared between the periods of applied or absent vibratory stimulus. Our results demonstrated that toe trajectory variability in the swing phase was significantly smaller when a vibratory stimulus was applied. Applying vibratory stimulus to the soles of the forefoot could potentially be used to reduce foot trajectory variability, which could reduce the risk of trips and associated falls during walking in older adults.

## Introduction

The risk of falls during walking is known to increase with aging^1^. Falls can cause serious injury and morbidities such as hip fractures and traumatic brain injuries^2,3^, leading to hospitalization and a bedridden state, with subsequent reductions in quality of life. Furthermore, falls due to tripping during level walking are reportedly a leading cause of death among older adults^4,5^. Therefore, the development of effective fall-prevention measures for older adults is imperative.

The risk of falls increases with age because of multifunctional problems such as motor control and visio-cognitive deficits. However, one direct cause of the increased risk of falls in older adults is attributed to increased foot clearance variabilities, representing the vertical distance between the tip of the foot of the swing leg and the walking surface^6–10^. The larger the variability in foot clearance, the higher the risk of the foot unexpectedly hitting the ground during the swing phase of the gait cycle, resulting in tripping, a major cause of falls during walking^6,9^. If the foot trajectory variability during the gait swing phase could be reduced, we could consequently reduce the risk of falls in older adults during walking.

Vibratory input to the sole of the feet has been indicated as an effective method of improving balance (postural stability) during quiet standing^11,12^. The detection of the somatosensory input signal may be enhanced by applying weak white-noise vibration. This phenomenon, called stochastic resonance, occurs when a noise signal is added to the somatosensory input, and the resulting sensory signal has a higher chance of exceeding sensory thresholds^13^. Consequently, vibratory input to the sole of the feet enhances the sensitivity of the cutaneous receptors in the sole, which are essential for the control of standing balance^14,15^, and the postural sway is reduced^11,12^. Recent studies have suggested that the vibratory input to the foot sole improves standing balance and reduces gait variability in older adults^16–19^. These studies reported that the use of vibrating insoles during walking significantly reduced the variability of temporal gait parameters such as gait cycle duration and stance and swing phase durations. Therefore, vibratory stimulation of the foot's plantar surface could be a potentially effective intervention for preventing falls in older adults during walking by reducing gait variability. However, no studies have yet investigated whether the vibratory stimulation of the foot's plantar surface can actually reduce foot trajectory variability and thus reduce fall risk during the swing phase of the gait cycle.

During normal human walking, the foot lands on the heel, and the center of pressure (COP) moves rapidly from the heel to the toes. Thus, the COP is located much longer (approximately two-thirds of the stance phase) in the anterior region of the sole (the ball of the foot) than in the posterior region^20^. Currently, the vertical ground reaction force reaches its second peak, and the anterior ground reaction force reaches its propulsive peak. The COP progresses to toe-off and the swing phase. Therefore, it is speculated that the anterior plantar sole is probably responsible for balance and gait control. Applying the vibratory input to the anterior region of the foot sole could improve the stability of the trunk segment in the stance phase and reduce the swing leg trajectory variability in the swing phase.

The present study aimed to investigate whether the foot trajectory variability could be reduced by applying vibratory input to the anterior region of the foot sole during walking in healthy adults. We tested the null hypothesis to determine if there was difference in the foot trajectory variability under vibratory stimulation application. Looking for the cause of change in the foot trajectory variability, we investigated the differences in the variabilities of spatiotemporal gait parameters and whole-body kinematics, which have reportedly been associated with the risk of falls during walking^21–23^.

## Methods

**Figure 1:**
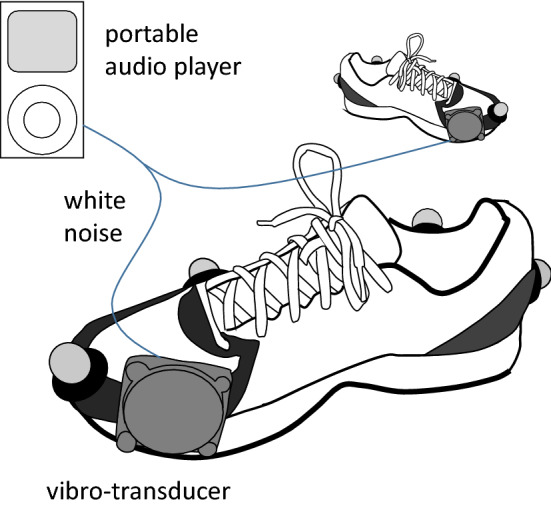
A pair of vibratory shoes constructed for the present study to apply a vibratory signal to the anterior region of the sole during walking.

A pair of vibratory shoes (Fig. 1) was developed to apply a vibratory signal to the anterior region of the sole during walking. We stitched a vibro-transducer Vp416 (Acouve Laboratory, Tokyo, Japan) to the anteromedial surface of an ordinary running shoe (JOG100-2; Asics, Kobe, Japan) using a fishing line. The transducer’s size was 56 mm × 56 mm × 17 mm, and its weight was 85 g. The vibrating element was placed outside of the shoe to avoid disrupting the insole construction and reduce the foreign body sensation in the shoe. Double-sided tape was used to enhance the attachment between the vibro-transducer and the sole. Since the shoe sole is much more rigid than the instep of the fabric shoe, the vibratory signal is transmitted to the shoe sole, allowing the vibratory signal to be firmly delivered to the plantar surface but not to the dorsum of the foot. A vibro-transducer was placed on both shoes. Both vibro-transducers were connected to a portable audio player (NW-A16; Sony, Tokyo, Japan) by a branch cable of producing mechanical vibration using an audio source of white noise. Thus, both feet received precisely the same synchronized vibratory signal. The white noise frequency range was 40–600 Hz, and frequencies < 40 Hz and > 600 Hz were attenuated. The amplitude of the noise was set at a constant value throughout the present study. By increasing and decreasing the amplitude, the value was set at a level that allowed the participant to be able just to feel and detect slight vibration in a quiet standing posture. Thus, the present study used as an input a supra-threshold, but not a sub-threshold vibratory signal.

**Figure 2:**
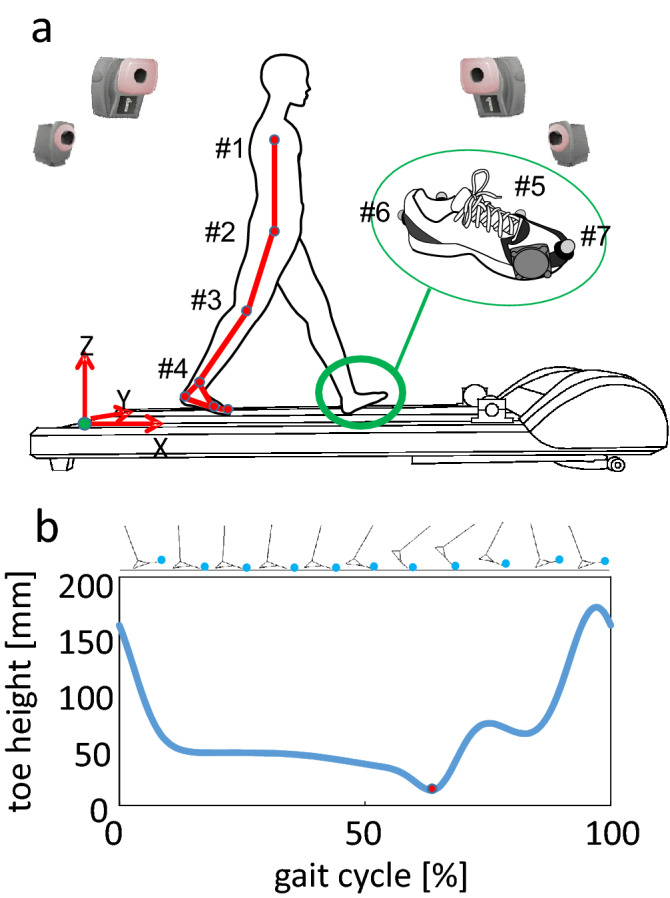
Schematic diagram of the experimental setup (**a**) and a representative profile of the height of the toe marker during walking (**b**). A total of 14 reflective markers (7 on each side) were attached to (1) the acromion, (2) the greater trochanter, (3) the lateral epicondyle of the femur, (4) the lateral malleolus of the fibula, (5) the head of the fifth metatarsal, (6) the calcaneal tuberosity, and (7) the toe (terminal phalanx of the third digit) to capture foot trajectory during walking. The dot indicates the time of toe-off.

Ten male university students (mean [± standard deviation] age, 22.7 ± 0.64 years; height, 1.68 ± 0.04 m; weight 57.1 ± 5.4 kg) without any history of orthopedic or neuromuscular impairments were enrolled to investigate whether the vibratory input to the anterior region of the sole reduced the foot trajectory variance. They were asked to walk on a 2.3-m long treadmill (D4017, ForceLink BV, Culemborg, Netherlands) set at 4 km/h (1.1 m/s) self-selected cadence while wearing the shoes with the vibro-transducers. The relatively slow treadmill speed was chosen to facilitate comparisons with older adults in future studies. The participants were provided with the same brand of running shoes of appropriate size. Body kinematics were recorded using a motion capture system consisting of 8 cameras (MAC3D; Motion Analysis Corporation, Santa Rosa, CA) (Fig. 2a). A total of 14 reflective markers (7 on each side) were attached to the acromion, greater trochanter, lateral epicondyle of the femur, lateral malleolus of the fibula, head of the fifth metatarsal, calcaneal tuberosity^24^, and toe (terminal phalanx of the third digit) to capture the foot trajectory during walking. The last four markers were placed on the corresponding surface positions of the shoes.

To adapt to the walking on the treadmill, we allowed participants to initially walk for 10 min as a practice session (the vibratory input was set to OFF). The walking gait was then recorded. The time was set to zero when the gait reached a steady state. The recording session was divided into two stages. The first was a 3-min OFF stage, with the vibratory input set to OFF. The second was a 3-min ON stage with the input set to ON. Body kinematics were captured at 200 Hz and low-pass filtered at 7 Hz^25^, using a second-order zero-phase shift digital low-pass filter. Data captured in the last 1-min of each stage were used for further analyses. Informed consent was obtained from all participants. The Ethics Committee of the Faculty of Science and Technology at Keio University approved this study. All methods were carried out following the relevant guidelines and regulations in the manuscript.

We calculated the cycle duration and stride length (the sagittally projected horizontal distance traveled in a gait cycle) using the motion-captured data. A gait cycle is defined as the period in which the foot initially contacts the ground to when the same foot contacts the ground again. The cycle duration was calculated by determining heel strike based on the height of the right heel marker. The stride length was calculated from the horizontal position of the right heel marker and the treadmill's velocity. Foot trajectory was defined as the change in the height of the right toe marker to the surface of the treadmill over time (Fig. 2b). Also, we calculated the hip, knee, and ankle joint angles of the right leg and trunk angle with respect to the vertical axis in the plane of progression (the x–z plane of the global coordinate frame). All joint angles are considered to be 0° in a quiet standing posture^26^. The trunk segment was defined as the segment connecting the midpoints of the left and right markers placed on the acromion and greater trochanter. For comparisons of foot trajectory and joint and trunk angles, time was normalized and expressed as a percentage of the cycle duration. All calculations were conducted using custom-made software written in C language.

Mean values for cycle duration, stride length, foot trajectory, and joint and trunk angles were calculated for each participant and condition (OFF and ON). To quantify variabilities in these gait parameters, we also calculated the mean absolute deviations (MADs) of these gait parameters. The MAD of the data {$x_{1} ,x_{2} , \ldots ,x_{n}$} can be calculated as:

1 $$ \Delta_{i} = \frac{1}{n}\sum\nolimits_{i = 1}^{n} {\left| {x_{i} - \overline{x}} \right|} $$

where $\Delta_{i}$ is the MAD of $x_{i}$, and $\overline{x}$ is the mean of the data. We used MAD instead of standard deviation and coefficient of variation because it is a more direct measure of gait variability. It uses absolute values of the differences between the data points and the mean, instead of squares of the differences, to circumvent the negative difference issue. Furthermore, it allows quantification of the parameters’ variabilities in every single step. Thus, we could also perform within-subject statistical comparisons of gait variabilities. A one-tailed paired t-test was performed to test for significant differences in means and MADs of cycle duration and stride length between the two conditions. The type I error rate (alpha) was set at 0.05. Normality and homogeneity of variance were tested using the Shapiro–Wilk test and Bartlett’s test, respectively.

To statistically compare the foot trajectory between the two conditions, means and MADs were calculated at a total of 10 time-points from 55 to 100% of the gait cycle corresponding to the swing phase. The foot trajectory during the entire swing phase was analyzed because the risk of falls could be related not only to the variability of the minimum foot clearance^9^. They could also be associated with the foot trajectory variability in the early and late swing phases, as the movements of the swing leg and trunk are determined by propulsive force generation in the late stance phase^23^, considering that the variability in the position of the heel contact (i.e., the change in the angle of attack) affects the stability of bipedal walking^27^. To compare joint and trunk angles, means and MADs were calculated at 10 time points at every 10% of the entire gait cycle. To test for significant differences in these parameters, multivariate analysis of variance (MANOVA) was performed. If MANOVA was significant, a one-sided, paired t-test was performed for each parameter, followed by Bonferroni correction for multiple testing with the adjusted p-value set at p < 0.005 (0.05/10). The Wilcoxon signed-rank test was used if tests for normality or homogeneity of variance failed. All statistical analyses were performed using R version 3.4.1 software^28^.

## Results

**Figure 3:**
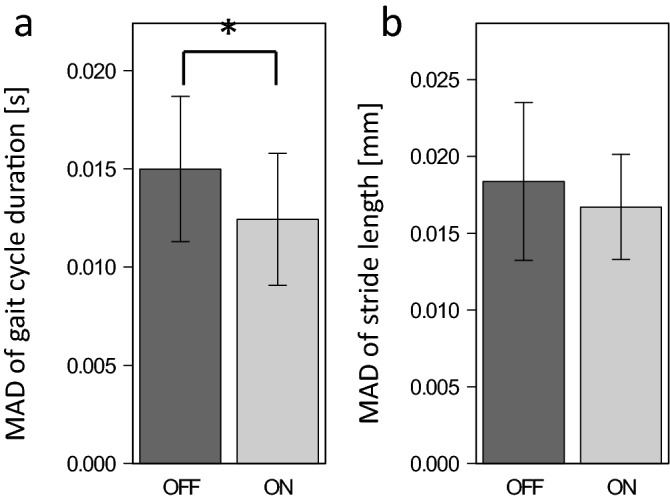
Comparison of the mean absolute deviations (MADs) of gait cycle duration (**a**) and stride length (**b**) between OFF and ON conditions. *p < 0.05.

Figure 3 compares the MADs of gait cycle duration and stride length between the OFF and ON conditions. Mean cycle duration and stride length were similar between the OFF and ON conditions: 1.10 ± 0.06 s and 1.10 ± 0.07 s, respectively for a cycle duration (t = 0.53, p = 0.70; tests for normality [W = 0.98 and 0.96, p = 0.94 and 0.79, respectively] and homogeneity of variance [Bartlett’s K-squared = 0.50, p = 0.48] were satisfied); and 1.22 ± 0.06 m and 1.22 ± 0.08 m, respectively, for stride length (t = 0.69, p = 0.75; tests for normality [W = 0.98 and 0.96, p = 0.95 and 0.81, respectively] and homogeneity of variance [Bartlett's K-squared = 0.48, p = 0.49] were satisfied). However, significant differences were detected in the MAD of cycle duration (t = − 2.62, p = 0.01; tests for normality [W = 0.94 and 0.91, p = 0.57 and 0.25, respectively] and homogeneity of variance [Bartlett's K-squared = 0.08; p = 0.77] were satisfied), thus, indicating that variability in cycle duration decreased with vibratory inputs. Significant differences were not observed for MAD of stride length (t = − 1.25, p = 0.12; tests for normality [W = 0.89 and 0.89, p = 0.19 and 0.18, respectively] and homogeneity of variance [Bartlett's K-squared = 1.36; p = 0.24] were satisfied).

**Figure 4:**
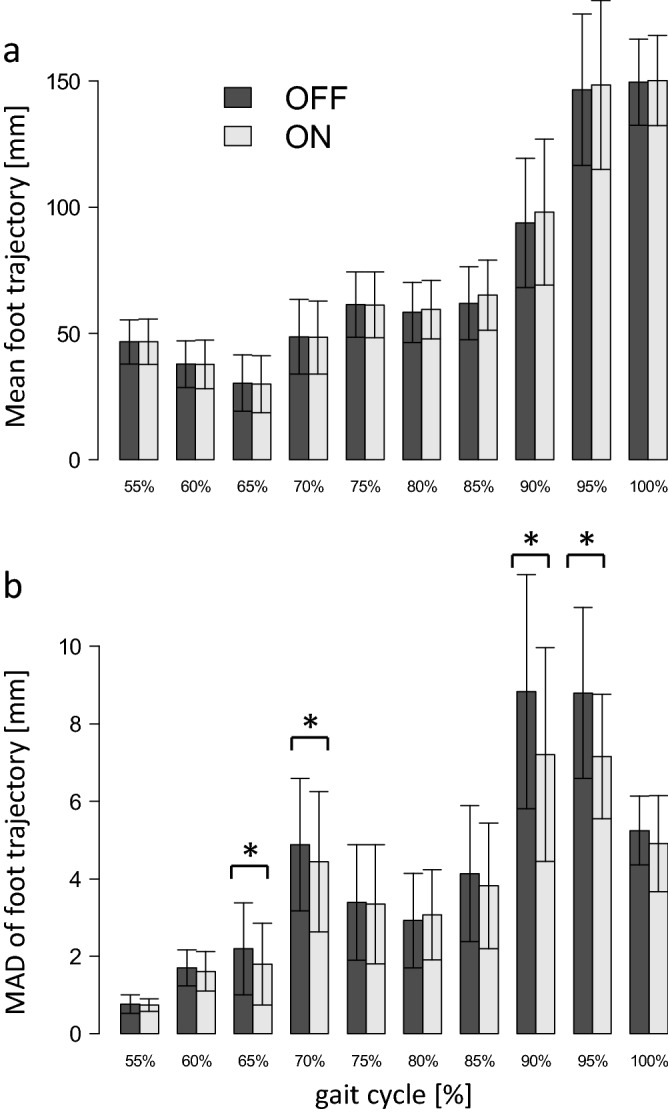
Comparison of the mean foot trajectory (**a**) and mean absolute deviations (MADs) of foot trajectory (**b**) during the second half of the gait cycle (swing phase) between OFF and ON conditions. *p < 0.005.

The mean foot trajectories calculated at 10 time points in the swing phase of the gait were virtually identical and not statistically different between the OFF and ON conditions [F(10, 9) = 1.10, Wilks' Lambda = 0.45, p = 0.45] (Fig. 4a). However, significant differences between the OFF and ON conditions were detected by MANOVA for MADs of the foot trajectory [F(10, 9) = 4.12, Wilks’ Lambda = 0.18, p = 0.02] (Fig. 4b). Multiple comparison revealed that MADs at 65% (1.8 mm vs. 2.2 mm; t = − 4.08, p = 0.001), 70% (4.4 mm and 4.9 mm; V = 3, p < 0.005), 90% (7.2 mm and 8.8 mm; t = − 4.88, p < 0.001), and 95% (7.2 mm and 8.8 mm; V = 0, p < 0.001) were significantly smaller in the ON condition than in the OFF condition. Thus, the variability of foot trajectory was reduced by the vibratory input, particularly in the early and late swing phases.

**Figure 5:**
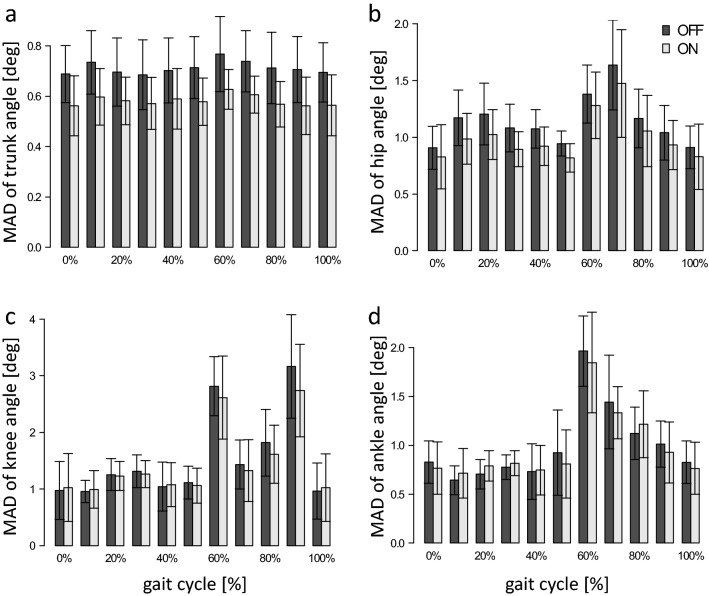
Comparison of the mean absolute deviations (MADs) of the trunk angle (**a**) and hip (**b**), knee (**c**), and ankle (**d**) joint angles between OFF and ON conditions.

Mean joint and trunk angles calculated at the 10 time points of the gait cycle were also identical and not statistically different between the OFF and ON conditions [F(10, 9) = 0.23, 0.68, 0.06, and 0.16; Wilks’ Lambda = 0.79, 0.57, 0.94, and 0.85; p = 0.98, 0.72, 1.00, and 1.00 for ankle, knee, hip joint, and trunk angles, respectively]. MAD of trunk angle tended to be smaller in the ON condition than in the OFF condition throughout the gait cycle (Fig. 5a). Although MANOVA indicated that the difference was not statistically significant [F(10, 9) = 2.91, Wilks’ Lambda = 0.23, p = 0.06], it was close to being statistically significant. On the other hand, changes in joint angle variabilities between the OFF and ON conditions were marginal (Fig. 5b–d) and not statistically significant [F(10, 9) = 0.83, 1.80, and 0.95; Wilks’ Lambda = 0.52, 0.33, and 0.49; p = 0.62, 0.20, and 0.54 for ankle, knee, and hip joints, respectively].

## Discussion

Vibratory input applied to the foot's plantar surface reduces the variability of some temporal gait parameters such as cycle duration^16,18^, as observed in the present study. However, our study's novel finding was that vibratory input reduces the foot trajectory variability during walking, improving this gait parameter, which is considered to be linked to the risk of falls during walking^6–10^. A previous study reported that phasic mechanical stimulation of the foot's plantar surface using pneumatic insoles during walking did not significantly affect the foot trajectory variability^29^. Therefore, vibratory input seems to be a key stimulation in reducing the foot trajectory variability during walking. Suppose the variability of the foot trajectory is large. In that case, the likelihood that the foot will unexpectedly hit the ground during the swing theoretically increases, leading to a higher chance of tripping and falling during walking^6,9^. The present study demonstrated that vibratory input to the foot's plantar surface could reduce the foot trajectory variability, positively associated with the risk of falls during walking.

The reduced foot trajectory variability is probably related to the body motion variability, particularly to trunk movement. It tends to be lowered when vibratory stimulation is applied to the stance leg. When the foot is in contact with the ground, a ground reaction force is applied to the foot's plantar surface, and cutaneous receptors of the plantar surface of the foot get activated. However, as no ground reaction force is applied during the swing phase, these receptors are much less activated. Therefore, cutaneous receptors of the plantar surface of the foot of the stance leg, but not in the swing phase, should play a critical role in successful control of standing balance^30^ and walking^31–33^. If sensory information from the cutaneous receptors of the stance leg is reduced, the ability to stabilize the standing balance is drastically hampered^34,35^. The present study suggested that vibratory input to the stance leg's plantar surface improved the static stability during quiet standing and the dynamic stability during walking. It results from enhancing the stance leg cutaneous receptors' sensitivity by applying vibratory input due to stochastic resonance. The foot trajectory variability during walking can be represented as the sum of the trunk orientation variability with respect to the inertial coordinate frame and the variability of the swing foot trajectory with respect to the trunk (hip joint). Therefore, if the vibratory input lowered the trunk movement's variability, the foot trajectory variability would also be reduced even though it did not change with respect to the hip joint. Hence, the variability of the trunk angle (i.e., postural sway), and consequently, that of the foot, as the distal end of the swing leg rotating about an imaginary axis fixed to the trunk, is reduced when vibratory stimulation is applied. There are reports that a stochastic resonance effect due to vibratory input to the foot's plantar surface enhances the cutaneous reflex to the leg muscles^36,37^. Such enhanced cutaneous reflex generation may also have contributed to the trunk's increased stability and reduced the foot trajectory variability.

A significant reduction in the foot trajectory variability was only observed at 65%, 70%, 90%, and 95% of the present study's gait cycle. The reason behind this is currently obscure, but it might be linked to comparatively larger acceleration, and deceleration of the foot segment observed at these points during walking ^38^. The detailed mechanism underlying the reduction in the foot trajectory variability due to vibratory input should be investigated in future studies.

In previous studies, sub-sensory vibratory stimulation was applied to the sole to improve balance during quiet standing^11,12^ and to reduce variabilities in temporal parameters during walking^16,18^. On the other hand, a previous study reported that supra-sensory vibratory input could have suppressed the sensitivity of foot sole receptors and possibly degraded postural control^39^ (but see Novak and Novak^40^ for an alternative argument). However, the present study applied a supra-sensory vibratory signal during quiet standing to reduce the foot trajectory variabilities. This is due to our concern that the sensory signal could be too weak during walking if it were sub-sensory during quiet standing. During quiet standing, the foot is axially loaded and thus sticks to the shoe sole, allowing better transmission of the vibratory signal from the shoe sole to the plantar surface of the foot. However, during walking, the transmission might have been degraded because the axial force and the tangential force were applied to the foot and the foot slightly moved in the shoe during walking. We asked the participants if they could feel the vibratory stimulation from the shoe sole during the walking experiment, and the response was it was hardly noticed by the participants during walking, although it was supra-sensory during quiet standing. Therefore, it was anticipated that the level was possibly quite similar between the supra-sensory signal used in the present study and the sub-sensory signal used in previous studies. This is perhaps why the vibratory input did not degrade but improved sensitivity and enhanced postural control during walking. Nevertheless, the way the change in the level of vibratory stimulation may improve or lessen postural stability during walking should be more rigorously investigated in future studies.

One limitation of the present study was that the participants were only young, healthy males whose gait variabilities should be much smaller than those of older adults^41^. The baseline foot trajectory variabilities measured in the present study were small, making it challenging to detect evident changes in the gait variabilities attributable to vibratory input. Additional validation studies should be planned to confirm the current findings in older adults and determine the present methodology's feasibility. Our preliminary measurements on one older adult showed that reductions in the foot trajectory variabilities and trunk and joint angles were more pronounced in older adults than in young, healthy males. This must be confirmed with larger-scale studies in the future. Another limitation is that we did not investigate how the input signal's location and strength applied to the foot's plantar surface affect the rate of decline in gait variability. Although we believe that the level and location of the vibratory input applied in the present study are appropriate, this must be confirmed in future research. The third limitation is that less foot trajectory variability could mean higher rigidity and inflexibility of gait, possibly leading to more frequent tripping^42,43^. The fourth limitation is that the order of the two conditions was not randomized, and it is not clear whether any time-varying effects confounded the present results. Although we believe these are not the case, such concerns must also be dispelled by further investigations.

In conclusion, the vibratory input to the foot sole could be an intervention for reducing foot trajectory fluctuations that are thought to be linked to the risk of falls during walking. Although further validation is necessary, the present approach could potentially be utilized to establish supportive technologies to reduce the risk of falls during daily walking in older adults.

## Data Availability

The datasets generated and/or analyzed during the current study and the custom-made software are available from the corresponding author upon reasonable request.
